# apoB/apoA‐I Ratio and Lp(a) Associations With Aortic Valve Stenosis Incidence: Insights From the EPIC‐Norfolk Prospective Population Study

**DOI:** 10.1161/JAHA.119.013020

**Published:** 2019-08-13

**Authors:** Kang H. Zheng, Benoit J. Arsenault, Yannick Kaiser, Kay‐Tee Khaw, Nicholas J. Wareham, Erik S. G. Stroes, S. Matthijs Boekholdt

**Affiliations:** ^1^ Department of Vascular Medicine Amsterdam Cardiovascular Sciences Amsterdam UMC University of Amsterdam Amsterdam The Netherlands; ^2^ Department of Cardiology Amsterdam Cardiovascular Sciences Amsterdam UMC University of Amsterdam Amsterdam The Netherlands; ^3^ Centre de Recherche de l'Institut Universitaire de Cardiologie et de Pneumologie de Québec ‐ Université Laval Québec Canada; ^4^ Department of Public Health and Primary Care University of Cambridge United Kingdom; ^5^ Medical Research Council Epidemiology Unit University of Cambridge Cambridge United Kingdom

**Keywords:** aortic valve stenosis, apoB/apoA‐I ratio, lipids and lipoproteins, lipoprotein(a), low‐density lipoprotein cholesterol, Cardiovascular Disease, Epidemiology, Risk Factors

## Abstract

**Background:**

Apolipoprotein B/apolipoprotein A‐I (apoB/apoA‐I) ratio and lipoprotein(a) (Lp[a]) are associated with aortic valve stenosis (AVS) disease progression. Clinical characteristics such as age, sex, and presence of concomitant coronary artery disease may strongly modify these associations; however, these effects have not been well defined in longitudinal studies. We set out to assess these associations between apoB/apoA‐I ratio, Lp(a), and AVS incidence in a large population study.

**Methods and Results:**

We analyzed data from 17 745 participants (mean age, 59.2±9.1 years; men, 44.9%) in the EPIC‐Norfolk (European Prospective Investigation Into Cancer in Norfolk Prospective Population Study) population study in whom apoB/apoA‐I and Lp(a) levels were measured. Participants were identified as having incident AVS if they were hospitalized or died with AVS as an underlying cause. After a median follow‐up of 19.8 years (17.9–21.0 years) there were 403 (2.2%) incident cases of AVS. The hazard ratio for AVS risk was 1.30 (95% CI, 1.19–1.41; *P*<0.001) per SD increase in apoB/apoA‐I. Adjusting for age, sex, and coronary artery disease, there was no significant association between apoB/apoA‐I and AVS incidence (hazard ratio, 1.06; 95% CI, 0.97–1.17 [*P*=0.215]). Elevated Lp(a) (>50 mg/dL) remained an independent risk factor for AVS after adjustment for age, sex, low‐density lipoprotein cholesterol, and concomitant coronary artery disease (hazard ratio, 1.70; 95% CI, 1.33–2.19 [*P*<0.001]).

**Conclusions:**

In this population study, apoB/apoA‐I ratio was associated with risk of AVS incidence, especially in younger and female participants and those without concomitant coronary artery disease. Lp(a) was an independent risk factor for AVS incidence. Interventional trials are needed to investigate whether modulating apoB/apoA‐I or lowering Lp(a) can prevent or slow down AVS.

Atherogenic apolipoprotein B (apoB)–containing lipoproteins such as low‐density lipoprotein (LDL) and lipoprotein(a) (Lp[a]) have clearly been implicated in the pathogenesis of aortic valve stenosis (AVS).^1^, ^2^However, trials aimed at reducing LDL cholesterol (LDL‐C) with statins and ezetimibe have failed to document a significant reduction in AVS progression^3^ and Lp(a)‐targeted trials have yet to be performed, currently leaving clinicians with no proven medical therapy to reduce disease progression.

A recent Swedish case‐control study reported that apoB/apolipoprotein A‐I (apoA‐I) ratio and Lp(a) were associated with future AVS surgery in patients with concomitant coronary artery disease (CAD) but not in those with “isolated” AVS.^4^ These findings suggested that patients with AVS could have different phenotypes, in which risk factors such as atherogenic lipoproteins may or may not play a major role in driving disease progression. In addition, post hoc analyses of the PROGRESSA (Metabolic Determinants of the Progression of Aortic Stenosis) trial^5^ found that apoB/apoA‐I was associated with faster hemodynamic progression in patients with mild to moderate AVS. These effects appeared especially pronounced in the younger subset of patients, suggesting that lipoprotein‐mediated pathology is dominant in younger patients, while other risk factors may contribute more to disease progression in elderly patients.

Risk factors for AVS progression can differ from those associated with AVS incidence as a result of underlying differences in pathophysiology in the initiation and propagation stages of disease.^6^ Large longitudinal cohorts are needed to assess whether and in whom these risk factors could be viable for intervention or risk stratification. It is unknown whether apoB/apoA‐I ratio is associated with AVS incidence in the general population. Whether the associations between apoB/apoA‐I ratio, Lp(a), and AVS incidence are independent of presence of CAD is also unknown.

The goal of this study was 2‐fold: (1) to assess the associations between apoB/apoA‐I ratio, Lp(a), and AVS incidence in a prospective, longitudinal study, and (2) to evaluate how clinical characteristics modify these associations. These data could have important implications for the design of future randomized trials to evaluate novel lipid‐lowering therapies, as well for individual risk assessment or treatment of patients with AVS.

## Methods

EPIC‐Norfolk (European Prospective Investigation Into Cancer in Norfolk Prospective Population Study) data will not be made publicly available for purposes of reproducing the results. Procedures to request access to EPIC‐Norfolk data can be found online (http://www.srl.cam.ac.uk/epic/).

### EPIC‐Norfolk Population Study

The EPIC‐Norfolk was a prospective population study of 25 639 male and female inhabitants of Norfolk, United Kingdom, aged between 39 and 79 years. The design and methods of EPIC‐Norfolk have been previously described in detail.^7^ Data were collected from February 1993 through March 2016. The primary outcome was AVS. Participants were identified as having incident AVS if they were hospitalized with AVS (*International Classification of Diseases, Tenth Revision* [*ICD‐10*] code I35) as an underlying cause or if they died with AVS as an underlying cause. We defined the presence of concomitant CAD as self‐reporting a history of prevalent CAD at baseline or first incident CAD during follow‐up (*ICD‐10* code I20–25). The Norwich District Health Authority Ethics Committee approved the study, and all participants gave signed informed consent.

### Laboratory Measurements

Conventional lipid profiles were measured at baseline as previously described.^7^ LDL‐C was calculated using the Friedewald formula. ApoB and apoA‐I were measured using rate immunonephelometry (Behring Nephelometer BNII). Lp(a) levels were measured in baseline samples (stored frozen at −80°C for ≈15 years) with an immunoturbidimetric assay using polyclonocal antibodies directed against epitopes in apolipoprotein(a) (Denka Seiken) as previously described.^8^

### Statistical Analyses

For this prospective analysis, study participants were excluded if apoB, apoA‐I, or Lp(a) levels were missing. Baseline characteristics and lipid measurements were compared between participants who developed AVS during follow‐up versus those who did not with the unpaired Student *t* test. Cox regression analysis was used to calculate hazard ratios (HRs) and corresponding 95% CIs for the time to hospitalization or death because of AVS. Statistical analyses were performed using SPSS software version 25 (SPSS Inc).

## Results

Complete data were available in 17 745 participants (mean age 59.2±9.1 years; male 44.9%) (Table). At baseline, 579 participants reported a history of CAD and 3300 participants had a first incident event of CAD during follow‐up. After a median follow‐up of 19.8 years (17.9–21.0 years), there were 403 (2.2%) incident cases of AVS. The associations between apoB/apoA‐I, Lp(a), and risk of incident AVS are shown in Figure.

**Table 1:** Baseline Characteristics

Variable	Controls	Cases	*P* Value
No. (%)	17 342 (97.8)	403 (2.2)	
Age, y	59.1±9.1	64.9±7.2	<0.001
Men, %	44.7	52.1	0.003
Body mass index, kg/m^2^	26.2±3.8	27.3±3.8	<0.001
Concomitant CAD, %	20.9	63.5	<0.001
LDL‐C, mg/dL	147±39	159±42	<0.001
apoA‐I, mg/dL	156±32	156±32	0.99
apoB, mg/dL	97±24	105±25	<0.001
apoB/apoA‐I ratio	0.64±0.19	0.70±0.19	<0.001
Lp(a), mg/dL	11.7 (6.3–27.7)	15.3 (7.0–41.7)	<0.001

Data are presented as mean±SD or median (interquartile range) unless otherwise indicated. Low‐density lipoprotein cholesterol (LDL‐C) was corrected for cholesterol content in lipoprotein(a) (Lp[a]): LDL‐C=LDL‐C−Lp(a) mass×0.3. ApoA‐I indicates apolipoprotein A‐I; apoB, apolipoprotein B; CAD, coronary artery disease.

**Figure 1:**
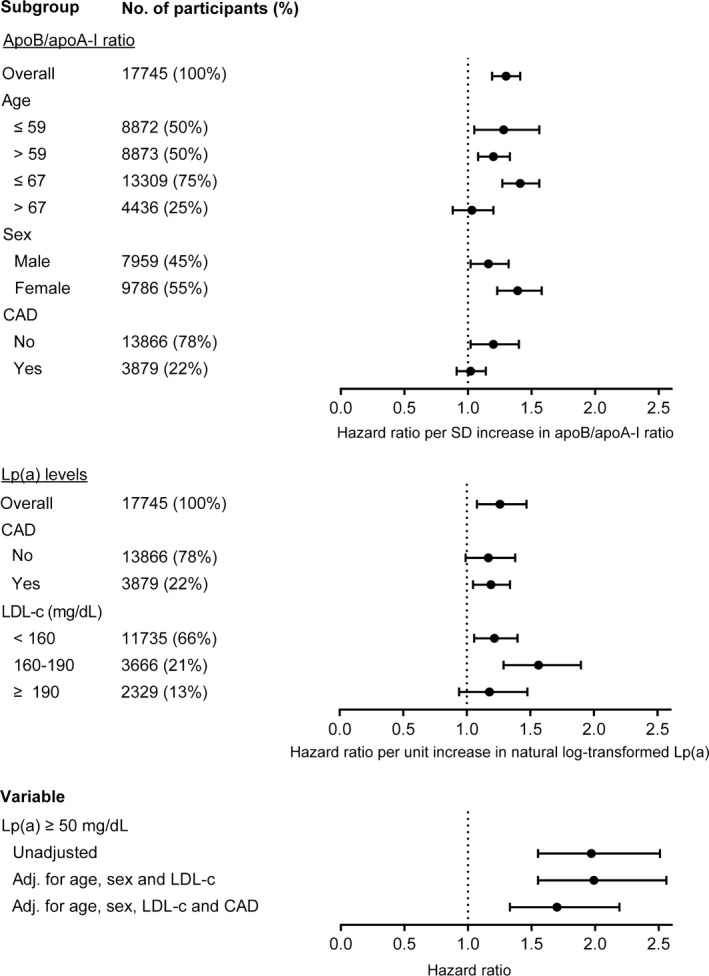
Risk of aortic valve stenosis (AVS) incidence associated with apolipoprotein B/apolipoprotein A‐I (apoB/apoA‐I) and lipoprotein(a) (Lp[a]). Multivariate Cox proportional hazards analysis for AVS incidence. CAD indicates concomitant coronary artery disease.

The mean apoB/apoA‐I ratio was higher among cases than among controls (0.70±0.19 versus 0.64±0.19; *P*<0.001). In the entire study cohort, the HR for AVS risk was 1.30 (95% CI, 1.19–1.41) per SD increase in apoB/apoA‐I. After adjusting for age and sex, this relationship was attenuated (HR, 1.18; 95% CI, 1.07–1.30 [*P*=0.001]). Notably, after additional adjustment for concomitant CAD, there was no significant association between apoB/apoA‐I and AVS incidence (HR, 1.06; 95% CI, 0.97–1.17 [*P*=0.215]). The risk of AVS stratified per apoB/apoA‐I quartile is documented in Figure S1.

We further investigated these findings by analyzing apoB/apoA‐I ratio and stratifying by age, sex, or concomitant CAD. In younger participants (median age, ≤59 years) the HR for AVS risk was 1.28 (95% CI, 1.05–1.56; *P*=0.014) for each SD increase in apoB/apoA‐I, whereas this appeared slightly lower in older participants (>59 years; HR, 1.20 [95% CI, 1.08–1.33]; *P*=0.001). In even older participants (>67 years, top quartile), the apoB/apoA‐I ratio was not associated with AVS risk (HR, 1.03; 95% CI, 0.88–1.20 [*P*=0.74]). Comparing sexes, we found a stronger association for apoB/apoA‐I ratio and AVS risk in women (HR, 1.39; 95% CI, 1.23–1.58 [*P*<0.001]) compared with men (HR, 1.16; 95% CI, 1.02–1.32 [*P*=0.025]). Stratifying for CAD, we found that apoB/apoA‐I ratio was associated with a higher risk in patients without concomitant CAD (HR, 1.20; 95% CI, 1.02–1.40 [*P*=0.025]), compared with participants with CAD (HR, 1.02; 95% CI, 0.91–1.14 [*P*=0.78]). These findings were further supported when we tested apoB/apoA‐I for interactions with age older than 67 years (*P*=0.001), sex (*P*=0.045), and concomitant CAD (*P*=0.075). Age as a continuous variable did not reach statistical significance as an interaction term (*P*=0.190).

In secondary analyses, we corrected apoB for its contribution in Lp(a). The associations between corrected apoB/apoA‐I and AVS incidence were slightly attenuated compared with uncorrected apoB/apoA‐I, but overall results were similar (Table S1, Figure S2).

Previously, we reported the independent associations between elevated levels of Lp(a) (≥50 mg/dL) and risk of incident AVS, after adjustment for age, sex, and LDL‐C.^9^ Additional adjustment for concomitant CAD did not markedly impact this relationship (HR, 1.99; 95% CI, 1.55–2.56 [*P*<0.001] versus CAD adjusted HR, 1.70; 95% CI, 1.33–2.19 [*P*<0.001]). Stratification by quartiles revealed that the risk for AVS clearly begins to increase in the top quartile (≥28 mg/dL) (Figure S1). The HRs per each unit increase of natural log‐transformed Lp(a) levels, stratified by the presence of concomitant CAD were also similar (no CAD HR, 1.17; 95% CI, 0.99–1.38 [*P*=0.06] versus CAD HR, 1.19; 95% CI, 1.05–1.34 [*P*=0.006]).

Elevated Lp(a) has been suggested to synergistically increase CAD risk in patients with elevated LDL‐C.^10^ To investigate this concept for AVS risk, we stratified for elevated LDL‐C and compared the HRs per each unit increase of natural‐log transformed Lp(a) (Table S2). Lp(a) levels appeared to confer more risk in patients with elevated LDL‐C between 160 mg/dL and 190 mg/dL (HR, 1.56; 95% CI, 1.29–1.90 [*P*<0.001]) compared with patients with LDL‐C <160 mg/dL (HR, 1.22; 95% CI, 1.06–1.40 [*P*=0.006]). However, this was not the case in patients with even higher LDL‐C ≥190 mg/dL (HR, 1.178; 95% CI, 0.940–1.477 [*P*=0.154]). We did not find a statistical significant interaction between corrected apoB/apoA‐I ratio and natural log‐transformed Lp(a) levels (*P*=0.916).

## Discussion

The apoB/apoA‐I ratio is considered to reflect the balance between atherogenic apoB‐containing particles and antiatherogenic high‐density lipoprotein particles.^11^ As such, this metric captures both dyslipidemia and dysmetabolic regulation. In our population study, apoB/apoA‐I ratio was found to be a strong predictor of AVS incidence, especially in younger and female participants. These findings support the concept that lipoproteins are involved in the pathogenesis of AVS, and are also compatible with the failure of previous statin trials to reduce AVS progression by LDL‐C lowering. Because these trials included mostly older (and male) patients with AVS, disease may have been too advanced to be amenable to lipid‐lowering therapy, suggesting that LDL or apoB‐containing particles play a predominant role in early stages of disease but are less important with increasing disease severity. Our observation that apoB/apoA‐I ratio was a stronger risk predictor in women compared with men is in line with emerging clinical data on sex‐related differences in AVS pathogenesis.^12^ Recent studies showed that women needed less valvular calcium burden than men to develop severe AVS,^13^ while women also demonstrated differential left ventricular remodeling and earlier symptom onset in AVS compared with men.^14^ Collectively, these data highlight the need for further studies to advance our understanding of sex differences in AVS pathogenesis to ultimately improve clinical management.

In contrast to an earlier report by Ljungberg and colleagues,^4^ we found that the apoB/apoA‐I ratio–associated risk of AVS incidence was higher in participants without concomitant CAD compared with those with CAD. Considering AVS shares many other risk factors with atherosclerosis, participants with CAD may also already have established subclinical AVS, at which stage increased apoB/apoA‐I may not confer any additional risk for AVS incidence. Interestingly, Lp(a) levels persisted as a risk factor in nearly all tested subgroups—including patients with concomitant CAD—suggesting that perhaps the oxidized phospholipids or other moiety on the Lp(a) particle act via different pathways than other apoB‐containing lipoproteins. These findings are in line with a recent multimodality imaging study in which elevated Lp(a) and oxidized phospholipids on apoB were associated with faster disease progression independent of concomitant CAD.^15^ Furthermore, a secondary analysis of the ASTRONOMER (Aortic Stenosis Progression Observation: Measuring Effects of Rosuvastatin) study,^16^ which excluded patients with concomitant CAD, demonstrated that elevated Lp(a) and oxidized phospholipids on apoB were associated with faster hemodynamic progression and need for valve replacement. In the setting of elevated LDL‐C, Lp(a) was associated with an additional risk of AVS incidence, although this was not clear in patients with severe hypercholesterolemia (LDL‐C ≥190 mg/dL). This group likely includes patients with a familial hypercholesterolemia phenotype, in whom AVS risk is increased such that the contribution of Lp(a) is relatively small, or perhaps that patients die of atherosclerotic disease before any AVS diagnosis can be made.

Clinical trials investigating novel therapies are needed to determine whether modulating the apoB/apoA‐I ratio or lowering Lp(a) can prevent or slow disease progress in AVS. Previous clinical studies have established that PCSK9 inhibitors are able to reduce both LDL‐C and Lp(a).^17^ In addition, the *PCSK9* R46L loss‐of‐function mutation is associated with reduced risk of AVS and myocardial infarction.^18^ Recently, experimental data suggested that PCSK9 may also directly facilitate calcification in valvular interstitial cells.^19^ Taken together, PCSK9 inhibition therapy could potentially reduce AVS disease progression and valve‐related events. To our knowledge, only 1 clinical trial is currently registered that aims to test this hypothesis (NCT03051360). Antisense oligonucleotides against apolipoprotein(a) have been demonstrated to effectively reduce Lp(a) levels,^20^ and a phase III cardiovascular outcomes trial is being planned. A dedicated antisense trial will be needed to test the Lp(a) hypothesis in patients with AVS.

### Study Limitations

Our study is observational and limited by defining AVS outcome based on *ICD‐10*–coded hospitalizations and mortality. In this respect, the diagnosis of AVS was not standardized. However, a large medical records study has recently reported that this *ICD‐10* approach resulted in high diagnostic accuracy (>90%) in Sweden,^21^ but also restricted outcome to moderate to severe AVS cases.

## Conclusions

In the EPIC‐Norfolk population study, apoB/apoA‐I ratio was associated with risk of AVS incidence, especially in younger and female participants and those without concomitant CAD. Lp(a) was an independent risk factor for AVS incidence.

## Disclosures

B. J. A. holds a junior scholar award from the *Fonds de recherche du Québec: Santé*. He has received research funding from the Canadian Institutes of Health Research, Pfizer, Merck, and Ionis Pharmaceuticals and is a consultant for Novartis. M. B. has served on the advisory boards of Pfizer and Sanofi. E. S. G. S.’ institution has received fees from lectures and advisory board participation from Amgen, Regeneron, Sanofi, Merck, Ionis, Chiesi, Akcea, Uniqur, and Athera. The remaining authors have no disclosures to report.

## Supporting information


**Table S1.** Baseline apoB Value Corrected for its Contribution in Lp(a)
**Table S2.** Risk of Aortic Valve Stenosis Associated With Natural Log‐Transformed Lp(a) Stratified by LDL‐C Levels
**Figure S1.** Risk of aortic valve stenosis incidence associated with quartiles of apolipoprotein B/apolipoprotein A‐I (apoB/apoA‐I) ratio and lipoprotein(a) (Lp[a]).
**Figure S2.** Risk of aortic valve stenosis incidence associated with corrected apolipoprotein B/apolipoprotein A‐I (apoB/apoA‐I).
