# Supplementary material for: apoB/apoA‐I Ratio and Lp(a) Associations With Aortic Valve Stenosis Incidence: Insights From the EPIC‐Norfolk Prospective Population Study
Source: J Am Heart Assoc. 2019 Aug 13;8(16):e013020. doi: 10.1161/JAHA.119.013020 (PMC6759902; doi:10.1161/JAHA.119.013020)
Supplement: Supplementary file 1 — Table S1. Baseline apoB Value Corrected for its Contribution in Lp(a) Table S2. Risk of Aortic Valve Stenosis Associated With Natural Log‐Transformed Lp(a) Stratified by LDL‐C Levels Figure S1. Risk of aortic valve stenosis incidence associated with quartiles of apolipoprotein B/apolipoprotein A‐I (apoB/apoA‐I) ratio and lipoprotein(a) (Lp[a]). Figure S2. Risk of aortic valve stenosis incidence associated with corrected apolipoprotein B/apolipoprotein A‐I (apoB/apoA‐I). [file JAH3-8-e013020-s001.pdf]

Supplemental Material

Table S1. Baseline apoB value corrected for its contribution in Lp(a).

Variable	**Controls**	**Cases**	**p-value**
Secondary analyses			
apoB corrected for Lp(a) mass	94 ± 24	101 ± 25	<0.001
$apoB_{corr.\ for\ Lp(a)\ mass}$/apoA-I ratio	0.62 ± 0.18	0.67 ± 0.19	<0.001

ApoB was corrected for its contribution in Lp(a) using the following calculation: apoB corrected for Lp(a) mass = apoB–(Lp(a)$_{mass}$*0.16]).[1]

Table S2. Risk of aortic valve stenosis associated with natural log-transformed Lp(a) stratified by LDL-c levels.

LDL-c group (mg/dL)	Unadj. HR (95% CI)	HR adj. for age and sex (95% CI)	HR adj. for age, sex and CAD (95% CI)
LDL-c < 160	1.217 (1.058-1.399); p=0.006	1.189 (1.031-1.379); p=0.017	1.131 (0.983-1.300); p=0.085
160 ≥ LDL-c < 190	1.564 (1.289-1.898); p<0.001	1.569 (1.284-1.916); p<0.001	1.444 (1.184-1.762); p<0.001
LDL-c ≥ 190	1.178 (0.940-1.477); p=0.154	1.130 (0.901-1.416); p=0.290	1.090 (0.870-1.365); p=0.453

HR = Hazard ratio per unit increase of natural log-transformed Lp(a) levels.

Figure S1. Risk of aortic valve stenosis incidence associated with quartiles of apoB/apoA-I ratio and Lp(a).

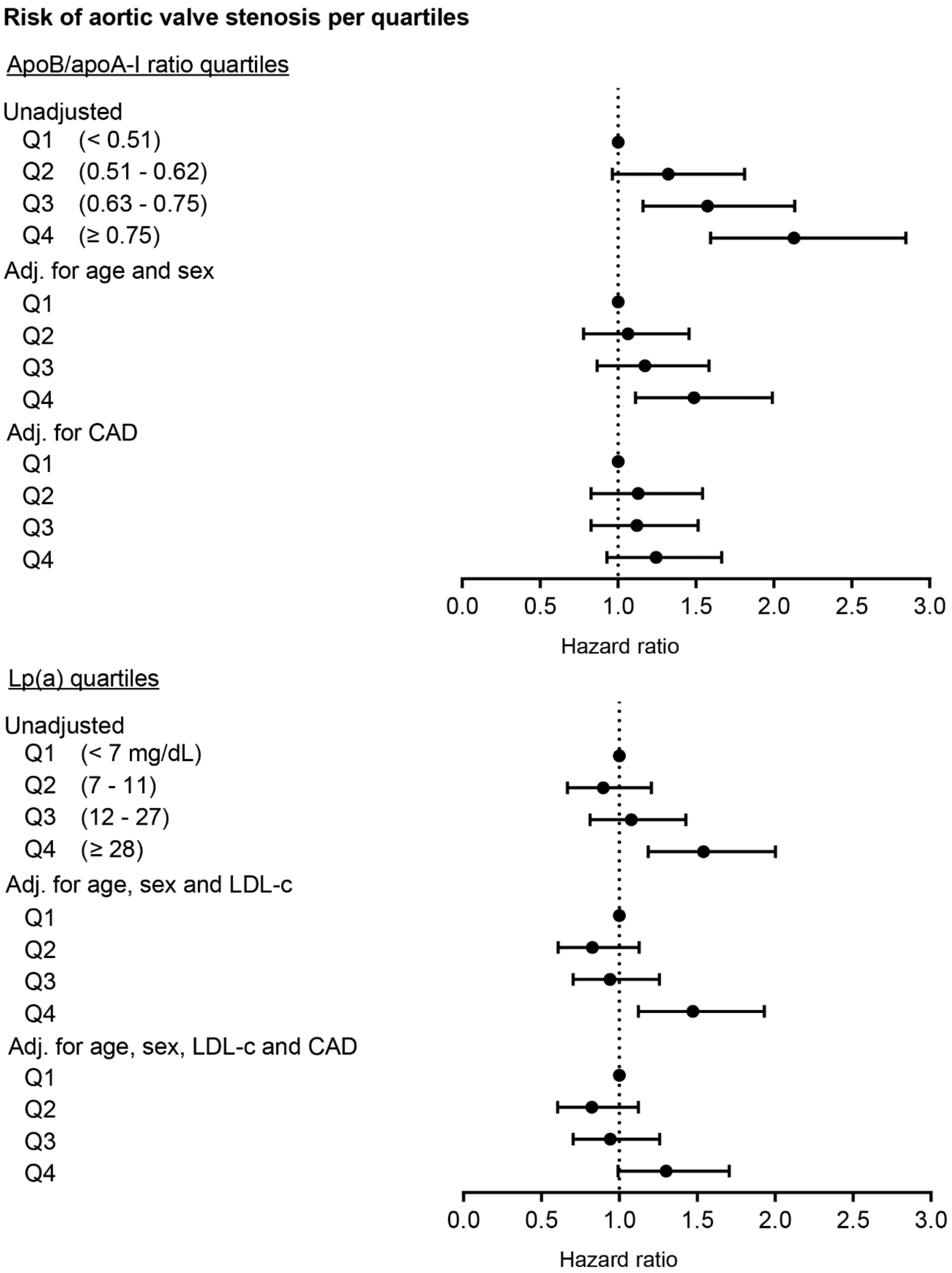

Multivariate Cox proportional hazards analysis for AVS incidence. Q = quartile; LDL-c = low-density lipoprotein cholesterol; CAD = concomitant coronary artery disease.

Figure S2. Risk of aortic valve stenosis incidence associated with corrected apoB/apoA-I.

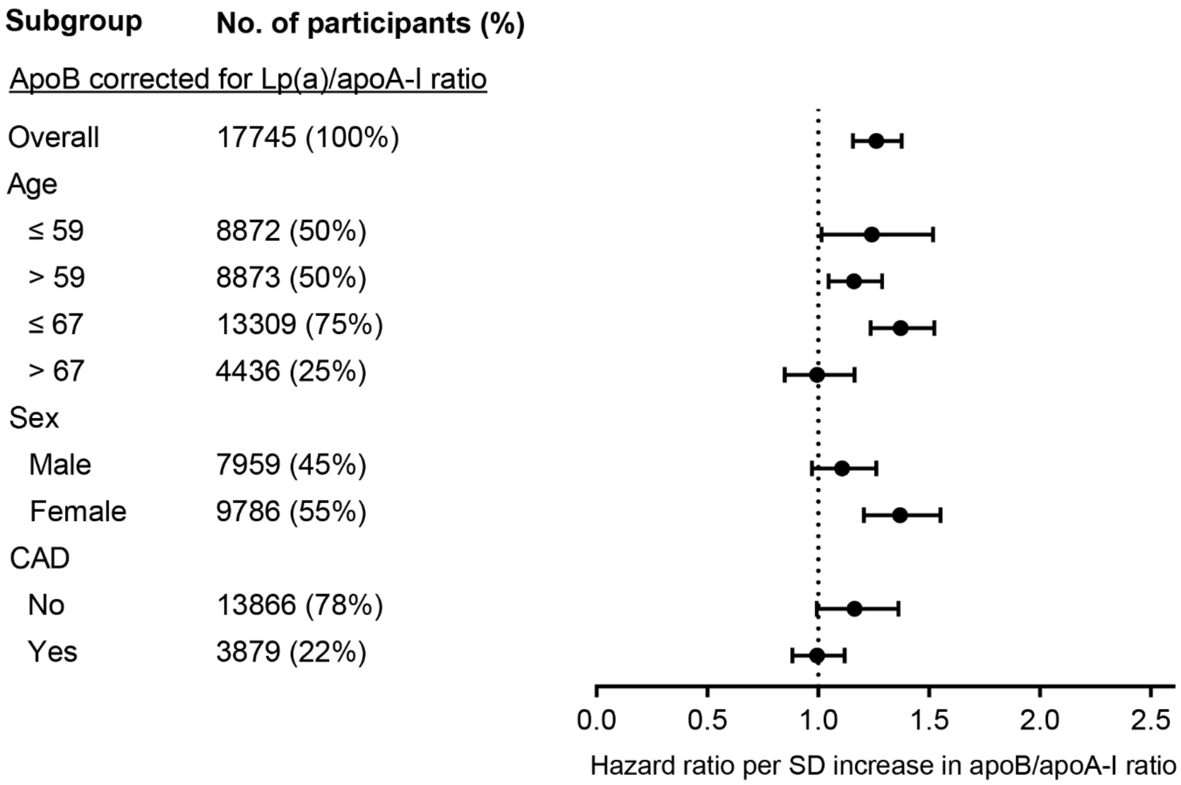

Multivariate Cox proportional hazards analysis for AVS incidence. CAD = concomitant coronary artery disease; SD = standard deviation.

Supplemental Reference:

1. Enkhmaa B, Anuurad E, Zhang W, Berglund L. Significant associations between lipoprotein(a) and corrected apolipoprotein b-100 levels in african-americans. *Atherosclerosis*. 2014;235:223-229.
